# Utility of prenatal trio whole-exome sequencing and methylation-specific multiplex ligation-dependent probe amplification in the evaluation of fetal growth restriction

**DOI:** 10.3389/fmed.2026.1741067

**Published:** 2026-06-05

**Authors:** Yangping Chen, Mianmian Wang, Xiaohe Cai, Xiuxiu Wu, Jiangzhong Zeng, Lili Zhou

**Affiliations:** 1 Department of Obstetrics and Gynecology, Wenzhou Central Hospital, Wenzhou, Zhejiang, China; 2 Center for Prenatal Diagnosis, Wenzhou Central Hospital, Wenzhou, Zhejiang, China

**Keywords:** fetal growth restriction, genetic etiology, MS-MLPA, prenatal diagnosis, trio-WES

## Abstract

**Objective:**

This study aimed to explore the application value of prenatal trio whole-exome sequencing (trio-WES) and methylation-specific multiplex ligation-dependent probe amplification (MS-MLPA) in fetal growth restriction (FGR).

**Methods:**

The clinical data of 66 cases of FGR in fetuses who underwent interventional prenatal diagnosis at Wenzhou Central Hospital from January 2022 to July 2025 were retrospectively collected. All fetuses had undergone chromosomal karyotype and CMA tests with negative results, and then, trio-WES was performed. Among the 21 cases with negative trio-WES results, MS-MLPA testing was further conducted. The *χ*^2^ test was used to analyze the abnormal rates of FGR across different groups.

**Results:**

In 66 cases of FGR, trio-WES could detect an additional 42.42% (28/66) of the abnormalities, among which likely pathogenic (LP)/pathogenic (P) was 27.27% (18/66). MS-MLPA detected two cases of abnormal methylation, with an abnormality rate of 9.52% (2/21).

**Conclusion:**

Trio-WES and MS-MLPA can improve the detection rate of genetic abnormalities in FGR. Trio-WES and MS-MLPA are recommended as further testing methods for FGR cases with negative CMA results.

## Introduction

FGR is a condition in which the intrauterine growth and development of a fetus fails to reach its genetic growth potential. It is mostly characterized by the estimated fetal weight or abdominal circumference being below the 10th percentile for the corresponding gestational age (1). FGR is one of the common and relatively complex complications during pregnancy, which can increase the rates of preterm birth and perinatal mortality. The etiologies of FGR include fetal, placental, and maternal factors, among which fetal genetic etiologies play a crucial role in the pregnancy outcomes of FGR (2). Two studies focusing on the genetic etiologies of FGR (3, 4) showed that the rate of chromosomal abnormalities ranged from 3.9 to 28.5%, and CMA could further increase the detection rate of abnormalities by 4.2 to 7.7%. However, the etiology of many cases of FGR remains unclear. Pauta et al. (5) reported that whole-exome sequencing could increase the diagnostic yield by 12% in cases of isolated FGR. According to the recommendations of the American College of Obstetricians and Gynecologists (ACOG) (6), prenatal diagnosis and counseling should be performed for fetuses with early-onset FGR or FGR complicated by structural malformations. Abnormalities of imprinted genes can lead to genetic syndromes such as Beckwith–Wiedemann syndrome (BWS), Temple syndrome, Silver–Russell syndrome (SRS), and transient neonatal diabetes mellitus (TNDM). These conditions involve multilocus imprinting disturbances, including hypomethylation at 11p15 (7) and 14q3 (8), maternal uniparental disomy (UPD) of chromosome (9), and abnormalities at the 6q24 locus (10). Among them, SRS, Temple syndrome, and transient neonatal diabetes mellitus (TNDM) can present with fetal growth restriction *in utero*. Multiplex ligation-dependent probe amplification (MLPA) is a novel method developed in recent years for detecting copy number variations (CNVs), which allows the analysis of multiple loci on different chromosomes in a single reaction. MLPA can be used for both genomic DNA and mRNA analysis. Methylation-specific multiplex ligation-dependent probe amplification (MS-MLPA) (11) is a modified MLPA technique designed to detect the methylation status of target DNA sequences. MS-MLPA has currently become the gold standard for the molecular diagnosis of genetic diseases caused by various DNA methylation abnormalities (12).

Nevertheless, there are limited studies evaluating the application of whole-exome sequencing (WES) or MS-MLPA technologies in FGR. In this study, 66 fetuses with FGR, who had previously undergone CMA with negative genetic results, were further subjected to trio-WES. Among the cases with negative results, an additional 21 cases were examined using MS-MLPA. This study aimed to explore the application value of these technologies in FGR.

## Subjects and methods

### Subjects

Demographic and clinical characteristics were collected from 66 pregnant women who received invasive prenatal diagnosis due to FGR in Wenzhou Central Hospital (Wenzhou, China) from January 2022 to July 2025. The pregnant women were aged between 20 and 43 years, and the gestational age at the time of trio-WES ranged between 22 and 33 weeks. The diagnostic criteria were as follows: for singleton pregnancies, the estimated fetal weight (EFW) or abdominal circumference (AC) evaluated by fetal ultrasound was below the 10th percentile (P10%) for the same gestational age or below the mean minus 2 standard deviations (M-2SD) for the same gestational age. The exclusion criteria were cases of FGR caused by maternal factors, such as chronic malnutrition, hypertensive disorders of pregnancy, placental dysfunction, or cytomegalovirus infection. According to the co-occurrence of FGR with other ultrasound abnormalities, the patients in this study were divided into four groups: the isolated FGR group (26 cases), the FGR group with a single ultrasound soft marker (12 cases), the FGR group with multiple ultrasound soft markers (15 cases), and the FGR group with ultrasound structural abnormalities (13 cases). Ultrasound soft markers are abnormalities detected during fetal ultrasound examinations that are not associated with the fetal structure. They are non-specific indicators and cannot definitively indicate fetal structural abnormalities, as they may represent normal variations. FGR patients were then divided into two groups according to maternal age: FGR with advanced maternal age and FGR without advanced maternal age. Finally, based on gestational age at FGR diagnosis, the groups were categorized as follows: <28 weeks, 28–31 weeks +6 days, and ≥32 weeks. Amniocentesis was performed at gestational ages of 18 to 26 weeks, and cordocentesis was performed after 26 weeks of gestational age. This study was approved by the Medical Ethics Committee of Wenzhou Central Hospital (L2024-02-006).

### Methods

Approximately 30 mL of amniotic fluid samples was collected during amniocentesis and transferred to two sterile Eppendorf (EP) tubes, and umbilical cord venous blood samples were collected and transferred to two blood collection tubes containing heparin and ethylenediaminetetraacetic acid (EDTA) anticoagulants, respectively.

### WES

If karyotyping and CMA results were negative, prenatal WES was performed to detect the exon region and neighboring intron regions of 7,099 genes in the human exome using a NextSeq 550Dx system (Illumina, Inc.), and all variants of established clinical significance were validated using Sanger sequencing. The pathogenicity of variants was interpreted following the guidelines for the interpretation of sequence variants recommended by the American College of Medical Genetics and Genomics (ACMG) and the Association for Molecular Pathology (13, 14), as well as the recommendations and documents from the Sequence Variant Interpretation Working Group,^1^ and the 2019 Guideline for Interpretation of Variant Pathogenicity recommended by the Association for Clinical Genomic Science (ACGS) and the British Society for Genetic Medicine.^2^ Variants were classified into pathogenic (P), likely pathogenic (LP), variant of uncertain significance (VUS), likely benign, and benign. Variants classified as P/LP as revealed by trio-WES were considered indicative of clinical significance.

### MS-MLPA

The MS-MLPA hybridization method and STR analysis were used to detect partial deletions, duplications, and methylation abnormalities at nine imprinted sites: 6q24.2 (PLAGL1 gene), 7p12.2 (GRB10 gene), 7q32.2 (MEST gene), 11p15.5 (H19 gene), 11p15.5 (KCNQ10T1 gene), 14q32.2 (MEG3 gene), 15q11.2 (SNRPN gene), 19q13.43 (PEG3 gene), and 20q13. The MS-MLPA kit (ME029) was supplied by MRC Holland (Netherlands), and the DNA sequencer (model 3500) was supplied by ABI (United States). All procedures were performed strictly according to the manufacturer’s instructions.

### Statistical analysis

All data were entered into Microsoft Excel 2021 (Microsoft Corporation; Redmond, WA, United States), and differences in proportions were tested for statistical significance using the chi-squared test. All statistical analyses were performed using the statistical software SPSS 25.0 (SPSS, Inc.; Chicago, IL, United States), and a *p*-value of <0.05 was considered statistically significant.

### Trio-WES and MS-MLPA results

#### Gene abnormality detected by trio-WES

Trio-WES achieved an additional detection rate of 42.42% (28/66) for abnormalities, with LP/P abnormalities accounting for 27.27% (18/66). The associated genes for growth and development were *IGF1R* (Case 1), *GNAS* (Case 11), *SBDS* (Case 17), and *SHOX* (Case 25). VUS was found in 10 (26.92%) fetuses, and two of these fetuses were identified with the growth- and development-related genes, including the *PORCN* gene (Case 6) and the *GH1* gene (Case 13) (Table 1).

**Table 1:** LP/P and VUS gene mutations detected by trio-WES and MS-MLPA among 30 cases with FGR negative for karyotyping and CMA.

Case No.	Maternal age	Gestational age at trio-WES /MLPA (weeks)	Prenatal ultrasound findings	Gene	Chromosome coordinates/bases/amino acids	Mutation type	Gene subregion	Type of variant	Inheritance mode	Associated syndrome	Gene mutation classification	Outcomes
1	28	31 weeks + 3 days	FGR (*p* < 3%)	IGF1R NM-000875.4	Chr15:99465356–99467933. ex.11-13del/p.?	HET	Exons 11–13	*De novo*	AD, AR	Insulin-like growth factor I, resistance to	LP	TOP
2	30	29 weeks + 5 days	FGR, FL, HL, and UL below the M-2SD line	DYNC2I2 NM-052844.4	Chr9 131397370 c.981+1G>A; Chr9 131418924 c.82-83insCA p.Ser28Thrfs*97	HET:HET	Intron 6: exon 1	Mat; Pat	AR, AR	Brachydactyly type 11 with or without polydactyly	LP	TOP
3	32	25 W + 5 days	FGR, FL, and HL below the M-3SD line	TRPS1 NM-014112.4	Chr8:116632278 c.47G>A p.Arg16Gln	HET	E:3/7	*De novo*	AD	Associated with autosomal dominant (AD) alopecia-nasal-finger syndrome type I [online Mendelian Inheritance in Man (OMIM): 190350] and type III (OMIM: 190351)	VUS	SGA
4	29	22 weeks	FGR	ZMYND11 NM-006624.7	Chr10 10:255928–255936	HET	Exon 3	Mat	AD	Autosomal dominant intellectual disability type 30 (OMIM: 105200)	LP	TOP
5	36	23 weeks + 3 days	FGR, FL, HL, and UL below the M-2SD line. Fetal left ventricular hyperecho	ICR1 methylation	11p15BWS/RSS region abnormality (low methylation)			*De novo*		Silver–Russell syndrome	P	TOP
6	24	24 weeks + 2 days	FGR, smaller kidneys, fetal microphthalmos	PORCN NM-203475.3	ChrX:48372996 c.929C>A p.Ser310Tyr	Het	Exon 10	Mat	XLD	Focal dermal hypoplasia (Goltz syndrome; OMIM: 300651), short stature, abnormal eye development (this is a male fetus)	VUS	TOP
7	32	31 weeks + 6 days	FGR, short limbs, ventricular septal defect, single umbilical artery	CTNNB1 NM-001904.4	Chr3:41266824 c.496-1G>A	Het	Intron 4	Pat	AD; SMu	(1) Exudative vitreoretinal disease type 7 (OMIM: 617572) is characterized by neurodevelopmental disorders with spastic diplegia and visual impairment (OMIM: 116806) (2) Colorectal cancer (OMIM: 114500) and hepatocellular carcinoma (OMIM: 114550), both somatic	LP	TOP
8	32	29 weeks	FGR, ventricular septal defect, femur, and HL below the M-2SD line	KCNJ2 NM-0008913.3	Chr17:68172183 c.1003C>T;p.His335Tyr	Het	Exon 2	*De novo*	AD.	(1) Short QT syndrome type 3 (OMIM: 609622), (2). Andersen syndrome, (OMIM: 170390), (3) Familial atrial fibrillation (OMIM: 613980)	VUS	TOP
9	34	26 weeks	FGR, BPD below the M-2SD line	ADGRV1 NM-032119.4	Chr5:89940546 c.2758C>T p.Arg920	Het	Exon15	Mat	AD; AR	Familial febrile seizures type 4; Usher syndrome type 2C	LP	Healthy full-term infant
10	32	32 weeks	FGR, BPD below the M-2SD line.	SQSTM1 NM-003900.5; GJB2 NM-004004.6	Chr5:179251038.c.486dupGp.His163Alafs*15; Chr13:20763612;c.109G>A. p.Va137Ile	SQSTM1 Het; GJB2 c.109G>A Hom	E:17/28 E:2/2	Pat; parents	AD; AR	Frontotemporal dementia and/or amyotrophic lateral sclerosis type 3, associated with autosomal recessive deafness type IA	LP; P	TOP
11	33	25 weeks + 2 days	FGR, oligohydramnios	GNAS NM-000516.5	Chr20:57480498. c.493C>T p.Arg165Cys	Het	E:6/13	*De novo*	AD	Pseudohypothyroidism, progressive bone dysplasia (OMIM: 103580; 603233): progressive osteomalacia (OMIM: 166350), associated with growth retardation and intellectual disability	P	TOP
12	33	33 weeks	FGR	SOX18 NM-018419.3	Chr20 62679698 c.976G>T p.Glu326	Het	Exon2	Pat	AD	Oligospermia-lymphedema-capillary dilation-nephrotic syndrome (OMIM: 137940)	VUS	Healthy full-term infant
13	42	25 weeks	FGR	GH1 NM-000515.5	Chr17 61995162 c.414C>A P.Asp138Glu	Het	Exon 4	*De novo*	AD	Isolated growth hormone deficiency type 2	VUS	TOP
14	31	23 W + 3 days	FGR, right kidney absent or underdeveloped, left kidney abnormally enlarged.	COL4A1 NM-001845.6	Chr 13 110813696 c.4483C>T P.Arg1495Cys	Het	Exon 49	*De novo*	AD	Hereditary vascular disease with nephropathy, aneurysm, muscle spasm, and cerebral leukoencephalopathy	VUS	TOP
15	43	27 weeks + 5 days	FGR, FL, and HL below the M-2SD line	ANK2 NM-OO1148.6	Chr4 114274902 c.5128G>T	Het	Exon 38	*De novo*	AD	(1) Long QT syndrome type 4 (2) Complex neurodevelopmental disorders	LP	TOP
16	30	28 weeks + 3 days	FGR, fetal veins from cysts	APOB NM-000384.3	Chr2 2129161 c.10579C>T P.Arg3527Trp	HET	Exon 26	Mat	AR, AD	(1) Familial type 2 low β lipoproteinemia (2) Familial hypercholesterolemia	P	Healthy full-term infant
17	40	30 weeks	FGR, FL, and HL below the M-2SD line	SBDS NM-016038.4	Chr7 66459273 c.184A>T P.Lys62	HET	Exon2	Fat	AR	Shwachman–Diamond syndrome 1 (OMIM:607444)	P	Healthy full-term infant
18	21	26 weeks + 2 days	FGR	CLCN1 NM_000083.3	Chr7 c.949C>T p.Arg317*	HET	Exon8	Mat	AD	Myotonia levior, myotonia congenita	P	TOP
19	30	22 weeks + 4 days	FGR, sinus hypoplasia	EVC2 NM-147127.5; NEK8 NM-178170.3	Chr4 5633572; Chr17 27064747	HET:HET	Exon 11; exon 6	Fat; mat	AD; AD	Weyers maxillary hypoplasia; polycystic kidney disease type 8	P; LP	TOP
20	24	25 weeks + 3 days	FGR, BPD below the M-2SD line, fetal bilateral renal echogenicity and left ventricular hyperechogenicity	SCN5A NM-198056.3	Chr3 38592932 c.4931G>A p.Arg1644His	HET	Exon 28	Fat	AD; AD	Long QT syndrome type 3, Brugada syndrome type 1	P	Healthy full-term infant
21	33	29 weeks + 5 days	FGR, the intra-abdominal segment of the umbilical vein is widened	BEST1 NM-004183.4	Chr11 61719298 c.20G>A p.Ser7Asn	HET	Exon2	Fat	AD	Yolk-like macular dystrophy type 2, retinitis pigmentosa type 50, vitreoretinal chorioretinopathy	LP	SGA
22	35	23 weeks	FGR, FL and HL below the M-2SD line	SCN5A NM-OO1099404.2	Chr3: 38622757 c.2893C>T p.Arg965Cys	HET	Exon17	Mat	AD	Yolk-like macular dystrophy type 2, retinitis pigmentosa type 50, vitreoretinal chorioretinopathy	LP	TOP
23	27	29 weeks	FGR	MYH3 NM-00247.4	Chr17 10543999 c.2170C>T	HET	Exon20	Mat	AD	1. Contracture, pterygium, and type 1A vertebral-wrist-tarsal fusion syndrome. 2. Distal joint contracture associated with type 2A and type 2B3.	LP	SGA
24	26	30 weeks + 5 days	FGR, single umbilical artery, aortic hypoplasia	ABCC8 NM-000352.6	Chr11 17426057–17430064	HET	Exons 23–28del	Fat	AD	(1) Non-insulin-dependent diabetes mellitus (2) Transient neonatal diabetes mellitus, type 2 diabetes mellitus, infants (3) Leucine sensitivity, hypoglycemia	LP	TOP
25	19	30 weeks + 3 days	FGR, FL and HL below the M-2SD line	SHOX NM-000451.4	ChrX 591526–612319	HET	Exon1-5Del;	Fat	PR, PD; AD	(1) Langer limb osteodysplasia. (2) Leri–Weill chondrodysplasia. 3. Familial idiopathic dwarfism	LP	TOP
26	24	26 weeks + 1 day	FGR, FL and HL below the M-2SD line	11p15 BWS/RSS region	IC2 hypomethylation			*De novo*	AD	BWS	P	TOP
27	24	30 weeks + 5 days	FGR	RHOBTB2 NM-001160036.2	Chr8 22864393 c.701G>A p.Arg234His	HET	Exon7	*De novo*	AD	Developmental and epileptic encephalopathy type 64	VUS	Healthy full-term infant
28	39	30 weeks + 2 days	FGR, BPD, and HC below the M-2SD line, multiple intracranial calcification foci	RNASEH2C NM-032193.4	Chr11 65487550 c.434G>T p.Arg145Leu; Chr11 65487523 c.461C>T P.Ala154Val	HET; HET	Exon 3; exon 3	Fat; mat	AR	Aicardi– Goutières syndrome 3	VUS	TOP
29	37	27 weeks	FGR	IGF1R NM-000875.5	Chr15 99442720 C.1117G>C p.Glu373Gln	HET	Exon 5	*De novo*	AD, AR	Insulin-like growth factor 1 resistance	VUS	SGA
30	33	21 weeks	FGR	WNK3 NM-020922.5	ChrX 54321041 c.1629-1638del p.Asp544leufs*41	HEMI	Exon 8	Mat	XLR	Pretorius syndrome	VUS	TOP

HOM, homozygous variant; HET, heterozygous variants; HEMI, hemizygous; Mat, maternal; Pat, paternal; AD, autosomal dominant; AR, autosomal recessive; XLD, X-linked dominant; XLR, X-linked recessive; TOP, termination of pregnancy; BPD, biparietal diameter; HC, head circumference; FL and HL, femur length and humerus length; UL, ulna length; SMu, segmental maternal uniparental disomy.

#### Gene abnormality detected by MS-MLPA

MS-MLPA can further increase the abnormal rate by 9.52% (2/21). In Case 5, ICR1 hypomethylation was observed, which may be associated with a methylation variation in this region that may contribute to Silver–Russell syndrome (SRS). Case 26 exhibited slightly lower IC1 methylation levels than normal controls (0.36 vs. 0.51, 0.34 vs. 0.53, 0.37 vs. 0.44), while IC2 exhibited significantly lower methylation (0.13 vs. 0.48, 0.16 vs. 0.55, 0.14 vs. 0.37, 0.12 vs. 0.48). The lower IC2 levels compared to IC1 indicate methylation abnormalities, suggesting that the fetus may be a Beckwith–Wiedemann syndrome (BWS) fetus. No abnormalities were observed in the remaining 19 cases.

#### Comparison of gene abnormality rates in different ultrasound abnormal groups by trio-WES

For the overall positive detection rate, FGR cases combined with multiple soft markers showed the highest abnormal rate of 66.67% (10/15), followed by the FGR combined with a single soft marker group (50%,6/12), the isolated FGR group (34.61%, 5/26), and the FGR combined with ultrasound structural abnormalities group (23.08%, 3/13). However, there was no statistically significant difference between the four groups (*p* > 0.05). There was a significant difference in the detection of LP/P gene abnormality among the four groups (*p* < 0.05) (Table 2).

**Table 2:** Comparison of abnormality rates in trio-WES results among 66 FGR cases with different ultrasound abnormalities.

No.	Group	Cases	Normal cases	Positive rate (%)^a^	Rate of gene mutation (%)	
					VUS	LP/P^b^
1	Isolated FGR	26	17	34.61 (9/26)	19.23 (5/26)	15.38 (4/26)
2	FGR complicated by a single ultrasound soft marker	12	6	50.00 (6/12)	0.00 (0/12)	50.00 (6/12)
3	FGR complicated by multiple ultrasound soft markers	15	5	66.67 (10/15)	13.33 (2/15)	53.33 (8/15)
4	FGR complicated by ultrasound structural abnormality	13	10	23.08 (3/13)	23.77 (3/13)	0.00 (0/13)
	Total	66	38	42.42 (28/66)	15.15 (10/66)	27.27 (18/66)

a No significant difference was found in the detection of gene abnormality among the four groups.(*χ*^2^ = 6.532, *p* > 0.05). Group 1 vs. Group 3, *χ*^2^ = 3.930, *p* < 0.05; Fisher’s exact probability test between Group 1 and Group 2, *p* = 0.481, *p* > 0.05; Fisher’s exact probability test between Group 1 and Group 4, *p* = 0.714, *p* > 0.05. Fisher’s exact probability test between Group 2 and Group 3, *p* = 0.452, *p* > 0.05. Fisher’s exact probability test between Group 2 and Group 4, *p* = 0.226, *p* > 0.05. Fisher’s exact probability test between Group 3 and Group 4, *p* = 0.03, *p* < 0.05.

b A significant difference was found in the detection of LP/P gene abnormality among the four groups (Fisher’s exact test; 37.5% expected frequencies <5. *p* = 0.001).

#### Comparison of gene abnormality rates between the advanced maternal age group and the non-advanced maternal age group by trio-WES

The abnormal detection rate of FGR in the advanced maternal age (AMA) group was 57.14%, compared with 38.46% in the non-advanced maternal age group, with no statistically significant difference (*p* > 0.05) (Table 3).

**Table 3:** Comparison of gene abnormality detection rates between the FGR combined with or without advanced maternal age group by trio-WES.

No.	Group	Cases	LP/P	VUS	Positive rate^a^ (%)	LP/P abnormality^b^ (%)
1	FGR + AMA (≥35 years)	14	5	3	57.14	21.43 (5/14)
2	FGR + non-AMA (<35 years)	52	13	7	38.46	28.85 (13/52)
	Total	66	18	10		27.27 (18/66)

AMA, advanced maternal age.

a No significant difference was found in the detection of gene abnormality between the two groups (*χ*^2^ = 1.576, *p* = 0.209, *p* > 0.05).

b The LP/P abnormality rates between the two groups by the corrected chi-squared test, *χ*^2^ = 0.212, *p* = 0.424, *p* > 0.05.

#### Comparison of gene abnormality rates in different gestational age groups by trio-WES

The detection rates of abnormalities among different groups were 46.15% (28 weeks-31 weeks + 6 days), 41.77% (<28 weeks), and 33.33% (≥32 weeks). No statistically significant difference was observed among the three groups (*p* > 0.05) (Table 4).

**Table 4:** Detection of gene abnormality across different gestational ages in FGR by trio-WES.

No.	Group	Negative case	Positive cases	Abnormal rate^a^ (%)
1	<28 weeks	20	14	41.77
2	28–31 weeks + 6 days	14	12	46.15
3	≥3 weeks	4	2	33.33

a Fisher’s exact test; 33.3% expected frequencies <5. *p* = 0.874.

#### Pregnancy outcomes

Among the 20 LP/P cases identified by trio-WES and MS-MLPA, 14 cases chose to terminate the pregnancy, 4 cases delivered appropriate-for-gestational age (AGA) infants, and 2 cases delivered full-term-small-for-gestational age (SGA) infants. Of the 10 VUS cases, 6 cases chose to terminate the pregnancy, 2 cases delivered SGA infants, and 2 cases delivered AGA infants. Among the 36 genetically normal cases, 29 cases delivered healthy AGA infants, 5 cases delivered SGA infants, and 2 cases delivered preterm infants (Table 5).

**Table 5:** Follow-up outcomes of 66 pregnancies.

Follow-up outcome		AGA	SGA	Preterm birth	Induced labor	Total
Negative genetic tests		29	5	2	0	36
Positive trio-WES	Likely pathogenic/pathogenic	4	2	0	12	18
	VUS	2	2	0	6	10
Positive MS-MLPA	Likely pathogenic/pathogenic	0	0	0	2	2
Total		35	9	2	20	66

AGA, appropriate for gestational age; SGA, small for gestational age.

## Discussion

In this study, trio-WES testing was performed on 66 cases with normal FGR karyotype and CMA results, resulting in an increased detection rate of abnormalities of 42.42%. Among these, clinically significant abnormalities (LP/P) accounted for 27.27%, which was slightly lower than the detection rate of 30.77%, as reported in our previous findings (15). Among the different ultrasound abnormality groups, the detection rate of trio-WES abnormalities was highest in the FGR combined with multiple soft markers group, but the difference was not statistically significant (*p* > 0.05). However, the LP/P abnormal rate was highest in the FGR combined with multiple soft markers group and showed statistically significant differences (*p* < 0.05). Case 1 was diagnosed with a novel *IGF1R* gene mutation with LP. Insulin-like growth factor-1 (IGF-1) regulates mammalian development, metabolism, and growth by binding to IGF1R (16). Patients with *IGF1R* gene mutations may develop severe prenatal and postnatal growth disorders, microcephaly, and developmental delays. In this case. Severe FGR was observed during intrauterine development, and the pregnant woman opted for pregnancy termination. Case 29 also showed *IGF1R* gene mutations, but at different loci, with a VUS pathogenicity classification. After clinical consultation, the pregnant woman opted to continue the pregnancy and finally delivered an SGA infant. Case 2 was found to carry a *DYNC2I2* gene complex heterozygous mutation inherited from both parents. The autosomal recessive mutation of the *DYNC2I2* gene is associated with type 11 short-pelvis thoracic hypoplasia, with or without polydactyly/polyneuropathy (17). Fetal ultrasound findings indicated short limbs and were consistent with the genetic phenotype. Case 4 exhibited a *ZMYND11* gene heterozygous mutation inherited from the mother, which may cause autosomal dominant (AD) intellectual disability type 30, characterized by delayed speech development and behavioral abnormalities (18). Other features may include various types of epileptic seizures, hypotonia, feeding difficulties, and craniofacial malformations. The pregnant woman had mild intellectual disability but no other clinical phenotypes. Case 6 exhibited a *PORCN* gene mutation, an X-linked gene inherited in an AD pattern. Its heterozygous mutation can cause focal dermal hypoplasia (FDH), a condition clinically characterized by skin abnormalities, ocular anomalies, facial deformities, abdominal wall defects, and short stature (19). Case 7 exhibited a heterozygous mutation in the *CTNNB1* gene, which may contribute to various cancers (20), retinal detachment, lens and vitreous opacity, growth retardation (21), and intellectual disability (22). The gene mutation was inherited from his father, who underwent retinal detachment surgery as a child and continues to have poor vision in both eyes. He was 160 cm tall and had no intellectual disability. Case 9 exhibited a mutation in the *ADGRV1* gene, in which the autosomal dominant variant is associated with familial febrile seizures type 4, while the recessive or heterozygous dominant variant is linked to Usher syndrome (23). Case 10 exhibited a heterozygous mutation in the *SQSTM1* gene (AD), which is associated with frontotemporal dementia and/or amyotrophic lateral sclerosis type 3 (24). The mutation was paternal, and the father currently shows no observable symptoms. The average age of onset for this disease is 63.5 ± 9.7 years. Case 11 was found to have a novel *GNAS* gene mutation, which may lead to pseudohypoparathyroidism (PHP) (25), with clinical manifestations including hypocalcemia, short stature, and ectopic ossification (26). Case 13 was identified with a novel heterozygous mutation in the *GH1* gene. Dominant mutations in the *GH1* gene may cause isolated growth hormone deficiency type 2 (IGD2), characterized by isolated FGR *in utero* (27). Case 17 was identified with a heterozygous mutation in the *SBDS* gene, where the biallelic pathogenic variation may lead to Shwachman–Diamond syndrome (SDS). SDS is a rare autosomal recessive genetic disorder, with 90% of cases associated with biallelic pathogenic mutations in the *SBDS* gene on chromosome 7q.11.21. As a ribosomopathy, SDS results from mutations in the SBDS gene that encodes a protein essential for ribosome maturation. Characteristic postnatal features include growth retardation, bone marrow failure, pancreatic exocrine insufficiency, and skeletal abnormalities. In this case, the mutation was inherited from the father, who exhibited no phenotypic manifestations, resulting in the delivery of a healthy full-term infant (28). Case 25 showed a deletion of exons 1–5 in the *SHOX* gene. While large gene segment deletions have been frequently reported, exonic deletions have only been documented in one previous case (29). The *SHOX* gene is a primary genetic cause of childhood short stature, with an incidence rate ranging from 1/1,000 to 1/2,000. The condition is characterized by short stature and/or limb skeletal abnormalities, including dwarfism and Madelung deformity (30). Autosomal dominant mutations in the *SHOX* gene are associated with Leri–Weill dyschondrosteosis. The father, who was 163 cm tall, was the source of the paternal mutation. The patient received growth hormone therapy for 2 years during adolescence. Case 28 was found to carry compound heterozygous variants in the RNASEH2C gene: c.434G>T (paternal) and c.461C>T (maternal). Both variants were classified as VUS in accordance with ACMG criteria, with an autosomal recessive inheritance pattern. Pathogenic complex heterozygous variant in this gene may also manifest as Aicardi–Goutières syndrome (31), presenting with cerebral atrophy, leukodystrophy, intracranial calcifications, and cerebrospinal fluid (CSF) lymphocytosis. The AGS phenotype may resemble intrauterine viral infections. The gene variant has been rarely reported, and according to the ACMG guidelines, it is currently classified as VUS with insufficient evidence of pathogenicity. However, it is strongly associated with fetal ultrasound phenotype and is therefore suspected to be pathogenic. The genetic analysis of the couple’s child’s peripheral blood confirmed a maternal c.461C>T mutation in the *RNASEH2C* gene, while MRI scans at the same time showed no significant abnormalities. A follow-up ultrasound scan at 34 weeks of pregnancy revealed fetal brain atrophy. The couple requested termination of the pregnancy. After deliberation by the Medical Ethics Committee, given the poor prognosis of the fetus, the couple’s reproductive rights were respected, and termination of pregnancy was approved. Case 30 was diagnosed with a heterozygous *WNK3* gene mutation (X-linked recessive, XLR), inherited from the mother (Mat), which may lead to Prader–Willi syndrome (OMIM: 300358). Prader–Willi syndrome is an XLR intellectual disability characterized by mild to severe intellectual disability, developmental delays, autism, or neuropsychiatric symptoms, often accompanied by varying degrees of language delays, epilepsy, and microcephaly (32). The fetus was male, and the pregnant woman had chosen to terminate the pregnancy. Five additional cases with LP/P variants inherited from either the father or the mother, which were unrelated to the primary phenotype, also underwent termination of pregnancy. These involved the *SQSTM1* and *GJB2* genes (Case 10), the *CLCN1* gene (Case 18), the *EVC2* and *NEK8* genes (Case 19), the *SCN5A* gene (Case 22), and the *ABCC8* gene (Case 24). Of the six cases with variants of VUS, two were associated with the FGR phenotype, involving the *PORCN* gene (XLD, mat) and the GH1 gene (AD, *de novo*), respectively. Although the pathogenicity was classified as VUS, all pregnant women ultimately chose to terminate the pregnancy due to aggravated FGR or concurrent structural abnormalities detected by ultrasound.

Whole-exome sequencing (WES) is a genomic analysis method that combines whole-genome exome capture technology with high-throughput sequencing to study exons or protein-coding regions. Exons, though accounting for only 1.5% of genomic DNA, contain approximately 22,000 genes (33). Regarding the genetic causes of fetal neurological abnormalities, Yaron et al. (34) noted that WES can increase the detection rate of abnormalities by 44% compared to CMA and recommended WES as the first-line diagnostic method for neurological developmental abnormalities. A meta-analysis by Mone et al. (35) demonstrated that WES can increase the detection rate of abnormalities by 4% in isolated FGR cases with negative chromosomal and CMA results, while increasing the detection rate by 30% in FGR cases with concurrent structural abnormalities detected by ultrasound. Our trio-WES detected a 42.42% incremental yield for the diagnosis of congenital abnormalities over CMA, slightly exceeding the literature. Imprinting genes (36), also known as genomic imprinting, refer to a phenomenon where specific genes or gene clusters in offspring are exclusively expressed by the paternal or maternal allele, while the allele from the other parent is silenced or minimally expressed. The maintenance, erasure, and reconstruction of gene imprinting are mostly achieved through epigenetic modification, with DNA methylation being the most common form. If the methylation modification of genes is abnormal, it will affect the growth and development of the fetus, which may cause intrauterine growth retardation or excessive growth. MLPA (37) is a novel method developed in recent years for detecting copy number variations, capable of identifying multiple chromosomal loci in a single reaction. MLPA can be used for both genomic DNA and mRNA detection. MS-MLPA is a type of MLPA used to detect DNA methylation at specific sequences. In Case 5, the fetus exhibited FGR and short limbs. Trio-WES showed no significant abnormalities. However, MS-MLPA (38) revealed a novel low methylation in the ICR1 domain of the 11p15BWS/RSS region. This low methylation variation in the region is associated with SRS (39, 40). SRS patients exhibit severe intrauterine growth restriction, postnatal developmental delays, and distinctive facial features. Case 26 showed no abnormalities on trio-WES. MS-MLPA analysis revealed slightly lower IC1 methylation levels (0.36 vs. 0.51, 0.34 vs. 0.53, 0.37 vs. 0.44) and lower IC2 methylation levels (0.13 vs. 0.48, 0.16 vs. 0.55, 0.14 vs. 0.37, 0.12 vs. 0.48) compared to normal controls. The lower IC2 levels relative to IC1 indicated methylation abnormalities, suggesting that the fetus may have BWS. BWS is an autosomal dominant growth disorder with a predisposition to tumor development. The condition exhibits clinical heterogeneity, with primary phenotypes including exophthalmos, macroglossia, omphalocele, and renal medullary cysts (41). The fetal genetic phenotype did not match the clinical phenotype. We conducted MS-MLPA testing on the umbilical cord blood, which yielded identical results to the amniotic fluid analysis. Subsequent ultrasound examinations revealed severe FGR and delayed brain development, leading to the termination of the pregnancy. Alhamdan and Shajira (42) reported a case of an extremely low birth weight preterm infant with FGR who developed macroglossia, left hemihypertrophy, umbilical hernia, and scalp hemangioma at 8 months of age and was diagnosed with Beckwith–Wiedemann syndrome (BWS). Although BWS is considered an overgrowth syndrome, with macrosomia being the predominant feature in 84% of cases (43), not all patients present with typical clinical manifestations. Case 26 in this study was also an atypical BWS patient, presenting with severe FGR *in utero*, which differs from the classic BWS phenotype.

Trio-WES improves the detection rate of genetic abnormalities, but the pathogenic classification and prognosis of the same gene may vary depending on its different loci. Both Case 1 and Case 29 exhibited IGF1R gene mutations, with the former being LP and the latter being VUS. The former terminated pregnancy for severe FGR, while the latter had a good neonatal outcome. When a gene mutation is classified as VUS, clinicians should evaluate the prognosis by considering both clinical manifestations and family history. Case 28 was found to carry a compound heterozygous mutation of c.434G>T (paternal) and c.461C>T (maternal) in the *RNASEH2C* gene. According to the ACMG guidelines, both variants were classified as variants of uncertain significance (VUS). The couple requested termination of the pregnancy. They were informed that the current VUS classification of the gene mutations could not serve as a sole basis for pregnancy termination. Considering that imaging had already indicated fetal brain atrophy, and following discussion by the Medical Ethics Committee, given the high risk of poor fetal prognosis suggested by the imaging findings, the couple’s wishes were respected, and pregnancy termination was approved. The genetic causes of FGR may include chromosomal and genetic abnormalities, as well as methylation modification abnormalities. The interpretation of the genetic report on FGR should be based on a comprehensive assessment that combines the gene’s pathogenicity, the family history of the pregnant woman, and the clinical phenotype to avoid missed diagnoses and misdiagnoses.

This study also has some shortcomings. First, cases of VUS in this article tended to result in pregnancy termination, and their prognosis could not be followed up. Second, the limited number of cases undergoing trio-WES and MS-MLPA profiling makes it impossible to fully evaluate their clinical value in FGR. Third, when a P/LP gene unrelated to the FGR phenotype is detected by trio-WES, should the couple be informed? These unexpected findings may lead to the termination of pregnancy. Fourth, prenatal WES differs from postnatal WES in depth and width. Prenatal WES has limited application scope and is highly dependent on fetal intrauterine phenotypes. Insufficient fetal phenotypic manifestations in utero commonly lead to underdiagnosis, which further increases the difficulty and complexity of prenatal genetic counseling.

## Conclusion

In summary, invasive prenatal diagnosis is recommended for fetuses with FGR, regardless of gestational age and whether soft markers are present, to rule out genetic abnormalities. Trio-WES combined with MS-MLPA can improve the diagnostic yield of genetic abnormalities in FGR. When karyotyping and CMA yield normal results in FGR cases, additional testing with trio-WES and MS-MLPA is advised to enhance the diagnostic rate, avoid missed diagnoses, reduce the incidence of birth defects, and alleviate family burden. Furthermore, for ongoing pregnancies involving fetuses with identified gene mutations, this approach can guide postnatal growth and developmental follow-up.

## Data Availability

The datasets presented in this article are not readily available because due to strict data management regulations of the hospital, raw research data are generally not allowed to be externally shared. Further inquiries can be directed to the corresponding author/s.

## Footnotes

1
https://www.clinicalgenome.org/working-groups/sequence-variant-interpretation/

2
https://www.acgs.uk.com/quality/best-practice-guidelines/
